# Decoding Niobium Carbide MXene Dual-Functional Photoactive Cathode in Photoenhanced Hybrid Zinc-Ion Capacitor

**DOI:** 10.1021/acsmaterialslett.3c01661

**Published:** 2024-03-08

**Authors:** Jalal Azadmanjiri, Jakub Regner, Jiri Sturala, Zdeněk Sofer

**Affiliations:** Department of Inorganic Chemistry, University of Chemistry and Technology Prague, Technická 5, 166 28 Prague 6, Czech Republic

## Abstract

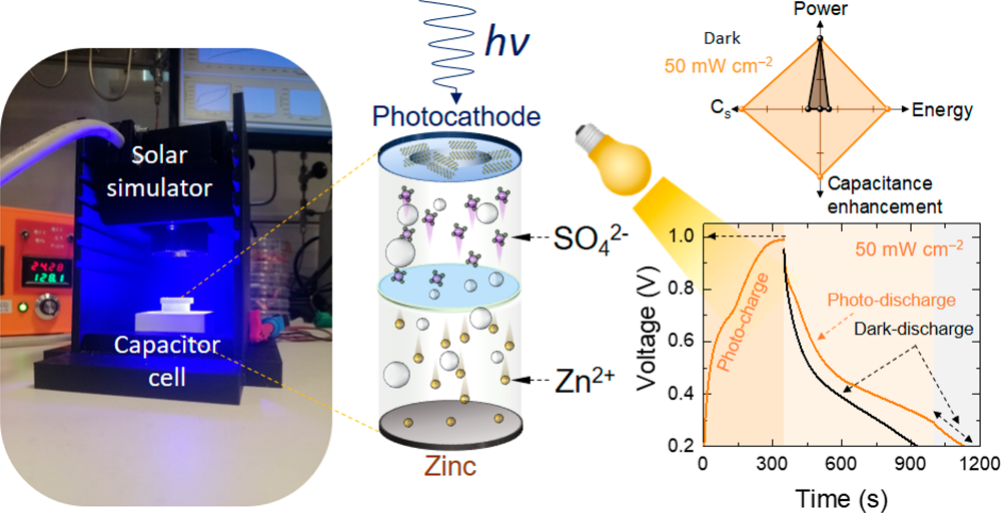

The coupling of energy harvesting and energy storage discrete modules in a single architecture as a “two-in-one” concept is significant in off-grid energy storage devices. This approach can decrease the device size and the loss of energy transmission in common integrated energy harvesting and storage systems. This work systematically investigates the photoactive characteristics of niobium carbide MXene, Nb_2_CT_*x*_, in a photoenhanced hybrid zinc-ion capacitor (P-ZIC). The unique configuration of the Nb_2_CT_*x*_ photoactive cathode absorbs light to charge the capacitor and enables it to operate continuously in the light-powered mode. The Nb_2_CT_*x*_-based P-ZIC shows a photodriven capacitance enhancement of over 60% at the scan rate of 10 mV s^–1^ under 50 mW cm^–2^ illumination with 435 nm wavelength. Furthermore, a photoenhanced specific capacitance of ∼27 F g^–1^, an impressive photocharging voltage response of 1.0 V, and capacitance retention of ∼85% (over 3000 cycles) are obtained.

Exploration of light–matter interactions, particularly in off-grid energy storage systems, has garnered considerable attention recently.^1,2^ This heightened interest is due to the recognition that light can either fully charge electrochemical energy storage systems or significantly develop their performances (i.e., quicker charging rates and capacitance enhancement).^3,4^ To have a robust architecture and highly efficient platform for a photoenhanced energy storage system, researchers are actively investigating materials possessing both simultaneously active optical and electrochemical properties.^5−7^ This dual functionality enables the light harvesting and storage of solar energy within the same material and components. Hence, numerous demonstrations for enhancing the performance of energy storage devices through light are under investigation in various energy storage systems, like lithium (Li)-ion batteries,^8−10^ Li–air batteries,^11,12^ and capacitors.^13,14^ Among them, there is a specific focus on the study of photoenhanced divalent zinc (Zn)-ion and magnesium (Mg)-ion batteries and capacitors as the most promising energy storage technologies^15−18^ because Zn and Mg anodes have unique features, such as the low cost of Zn^19^ and the natural abundance of Mg,^20^ superior safety,^21,22^ high specific capacity of 820 mAh g^–1^ and 5851 mAh cm^–3^ for Zn^23^ and 2205 mAh g^–1^ and 3833 mAh cm^–3^ for Mg,^24^ low redox potential (*E*_Zn_= −0.76 V vs standard hydrogen electrode),^25^ and long-term stability.^26,27^ Nevertheless, the kinetics of Mg-ion energy storage systems tend to be sluggish compared with monovalent ions like Li.^16^ Hence, this work presents a groundbreaking effort on a hybrid Zn-ion capacitor with 2D niobium carbide (Nb_2_CT_*x*_) MXene as a dual-functional photoactive electrode material capable of driving light enhancement while at the same time storing charges. The hypothesis of Nb_2_CT_*x*_ MXene selection is because of its narrow band gap (∼0.81 eV) belonging to the transition metal carbide core (Nb_2_C),^28^ and the band gap value of ∼1.3 eV mainly comes from the formation of a niobium dioxide (NbO_2_) surface layer.^29^

These two phases make the Nb_2_CT_*x*_ MXene a powerful nanostructure electrode material for simultaneous light harvesting and charge storage. Figure 1a–d indicates the crystal phase, structure, and morphology of the Nb_2_CT_*x*_ MXene characterized using X-ray diffraction (XRD), Raman spectroscopy, scanning electron microscopy (SEM), and energy-dispersive X-ray spectroscopy (EDS). It has been demonstrated by XRD that aluminum (Al) layers of the Nb_2_AlC MAX phase were almost completely etched during the synthesis of Nb_2_CT_*x*_ with only one remaining moderate peak at 2θ = 12.8° corresponding to starting MAX phase, which resulted in a multilayer structure of Nb_2_CT_*x*_. Raman characterization demonstrates the vibrational modes of Nb_2_CT_*x*_ at the six main Raman shifts specified in Figure 1b. SEM and EDS of the Nb_2_CT_*x*_ confirm the layered structure of the sample, including a trace of Al, as well as −O and −F terminals (Figure 1c,d). Figure 1e depicts an optical image (inset), SEM, and EDS of the Nb_2_CT_*x*_-based slurry that was synthesized by carbon black and PVDF. The slurry was synthesized for the next steps to determine its localized electrochemical reactivity, optoelectronic properties, and electrochemical behavior of Nb_2_CT_*x*_ as a photocathode material. Figure 1f,g displays 3D topographic representations of the current distribution near the surface of the Nb_2_CT_*x*_-based slurry mixture. The figures include values for roughness (*z*) and current at the tip of the ultramicroelectrode (UME). Both the roughness (geometry) and physical properties (i.e., conductivity) of the material influence the measured current in feedback mode. When the UME is positioned near the surface of an insulating material, the hemispherical diffusion layer is obstructed, which results in a reduction of current. Conversely, as the UME approaches a conductive (or electrochemically active) surface, despite the hindered hemispherical diffusion layer, the current increases because of the regeneration of the mediator within the electrolyte.^30^ Since the contribution of the roughness to the overall measured current is negligible, the obtained positive feedback currents in scanning electrochemical microscopy measurement of Nb_2_CT_*x*_ in the designated area are caused by the conductive properties of the sample and substrate. The obtained current values of the dried slurry made by the Nb_2_CT_*x*_ sample inside of the cavity are mainly similar in magnitude to those of the SiO_2_ substrate surrounding the sample. Nonetheless, few peaks of measured current were observed (four highlighted red circles in Figure 1h) over the surface of the slurry indicating increased regeneration of the Fe(CN)_6_^3–^ + e^–^ → Fe(CN)_6_^4–^ redox reaction in comparison with the SiO_2_. These points also were confirmed by SEM and EDS analyses.

**Figure 1:**
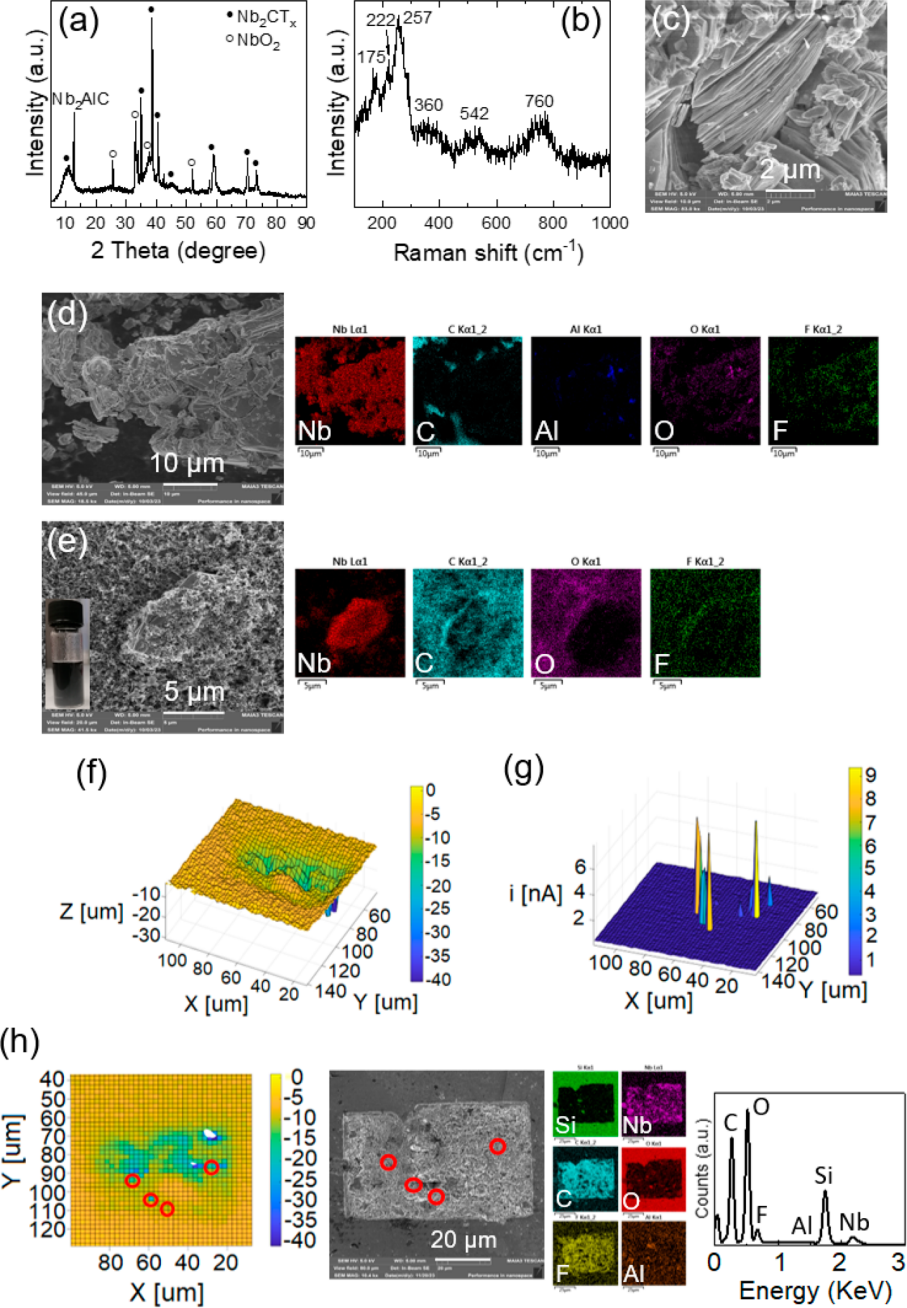
(a) XRD pattern, (b) Raman spectrum, and (c,d) SEM and EDS analyses of the synthesized Nb_2_CT_*x*_ MXene powder. (e) SEM, optical image (inset), and EDS analyses of the Nb_2_CT_*x*_-based slurry. (f–h) 3D topographical mapping catalytic activity of drop-casted Nb_2_CT_*x*_-based slurry, roughness (*z*), and current value, as well as SEM and EDS analyses of the examined four circle points indicated on 2D topographical mapping.

To evaluate the optoelectronic and initial electrochemical characteristics of the Nb_2_CT_*x*_-based photocathode, a self-powered photodetector device was set up in a beaker cell using a three-electrode system (Figure 2a). To do so, as shown in Figure 2a, around 10 μL of the as-prepared Nb_2_CT_*x*_-based slurry was drop-casted on a flexible and transparent polyethylene terephthalate-coated indium tin oxide (∼80 nm) and gold (∼20 nm) (PET-coated ITO/Au) substrate. Then, a drop of Nafion (C_7_HF_13_O_5_S·C_2_F_4_) was deployed on top of the electrode to form a stable structure in the following tests. The optoelectronic and electrochemical efficiencies of the Nb_2_CT_*x*_-based photocathode were tested in both dark and illumination conditions by an LED with λ = 435 nm and an intensity of 100 mW cm^–2^. Figure 2b demonstrates the energy band diagram and energetically favorable passage for the photogenerated electrons in the Nb_2_CT_*x*_-based photocathode after illumination.

**Figure 2:**
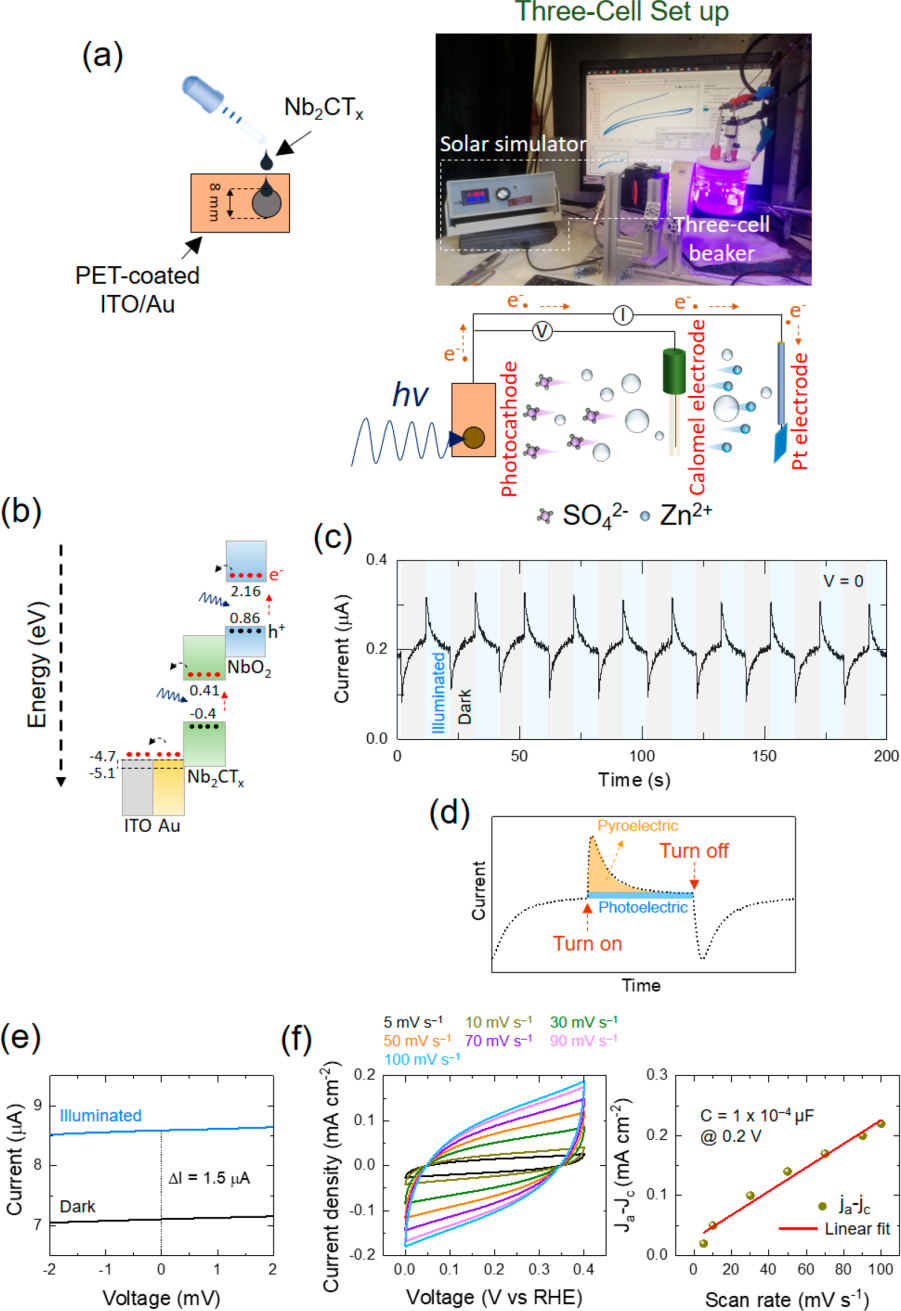
(a) Schematic illustration of the preparation of Nb_2_CT_*x*_-based photocathode constructed on PET-coated ITO/Au substrate and the three-electrode cell setup. (b) Schematic demonstration of the energy band diagram and energetically favorable trajectory of the prepared Nb_2_CT_*x*_-based photocathode. (c) Current–time plot with repeated dark and illumination conditions at 0 applied voltage. (d) Schematic illustrates the generated current versus time because of two pyroelectric and photoelectric effects under illumination and dark pulses. (e) Current–voltage plots under dark and illumination conditions at a low scan rate of 5 mA s^–1^. (f) Cyclic voltammogram at different scan rates and evaluation of the double-layer capacitance plot of the Nb_2_CT_*x*_-based photocathode under dark conditions.

It can be seen that both Nb_2_C and NbO_2_ possess lower band gap values than the energy of the illuminated λ = 435 nm light with ∼2.85 eV. As a result, photogenerated electrons could drift from NbO_2_ and Nb_2_C toward PET-coated ITO/Au through additive carbon black and propagate in the system. Therefore, the separated electrons and holes tend to adsorb the hydrated Zn^2+^ cations and SO_4_^2–^ anions of the ionized ZnSO_4_ aqueous electrolyte. The current–time plot of the three-cell system under periodic illumination at zero applied voltage is shown in Figure 2c. The current signal exhibits an immediate increase upon exposure to light, followed by a decrease upon turning off the light. Notably, there is an absence of a steady-state current during both illuminated and dark periods. This recognized behavior in current is ascribed to the pyroelectric effect (Figure 2d), probably because of the NbO_2_ phase in the MXene sample.^31^ However, the photocurrent gradually stabilizes over time and reaches a constant value. This stabilization is primarily due to the diminishing pyroelectric polarization potential caused by a steady-state temperature (d*T*/d*t* = 0, *T* = temperature, *t* = time). A similar pattern is also observed upon switching off the LED light. The linear sweep voltammetry (LSV) characteristic of the photocathode was performed in dark and illumination conditions to further assess the photocurrent behavior. Figure 2e illustrates a higher current value over the all-applied voltage range under illumination compared with that under the dark condition. This activity indicates the optimal photosensitivity behavior of the Nb_2_CT_*x*_-based photocathode for the generation and separation of electrons and holes with a result in the formation of a higher photocurrent. The generated photocurrent by the Nb_2_CT_*x*_-based electrode in chronoamperometry characterization (∼0.2 μA) is different from LSV (∼1.5 μA). This difference in photocurrent could be due to a higher level of stability and constancy of the current response over time by chronoamperometry in comparison with LSV. The current response stability by chronoamperometry arises from the fact that the applied voltage is constant throughout the experimental procedure and this keeps the material stable within the test. Nevertheless, the applied voltage fluctuates during LSV analyses, with an electrochemical process occurring on the electrode material within the applied voltage range, which renders the electrode less stable. Thus, the different current responses recognized by these two techniques can be ascribed to the distinct measurement parameters and the various electrochemical processes taking place on the electrode material. The energy storage capability of the Nb_2_CT_*x*_-based photocathode was also determined through the three-electrode system. This was done by the CV responses at various scan rates within the working voltage of 0.0 to 0.4 V (Figure 2f). The selection of this relatively low working voltage range was due to blocking of any gas formation. Notably, the current density reaches 0.2 mA cm^–2^ at a scan rate of 100 mV s^–1^, while the double-layer capacitance attains a value of 1 × 10^–4^ μF at 0.2 V. In the next step, the as-prepared Nb_2_CT_*x*_-based photocathode was assembled inside a printed holder with an optical window of 8 mm diameter as a photoenhanced hybrid zinc-ion capacitor (P-ZIC) cell to evaluate its energy storage mechanism and performance under dark and illumination conditions (Figure 3a).

**Figure 3:**
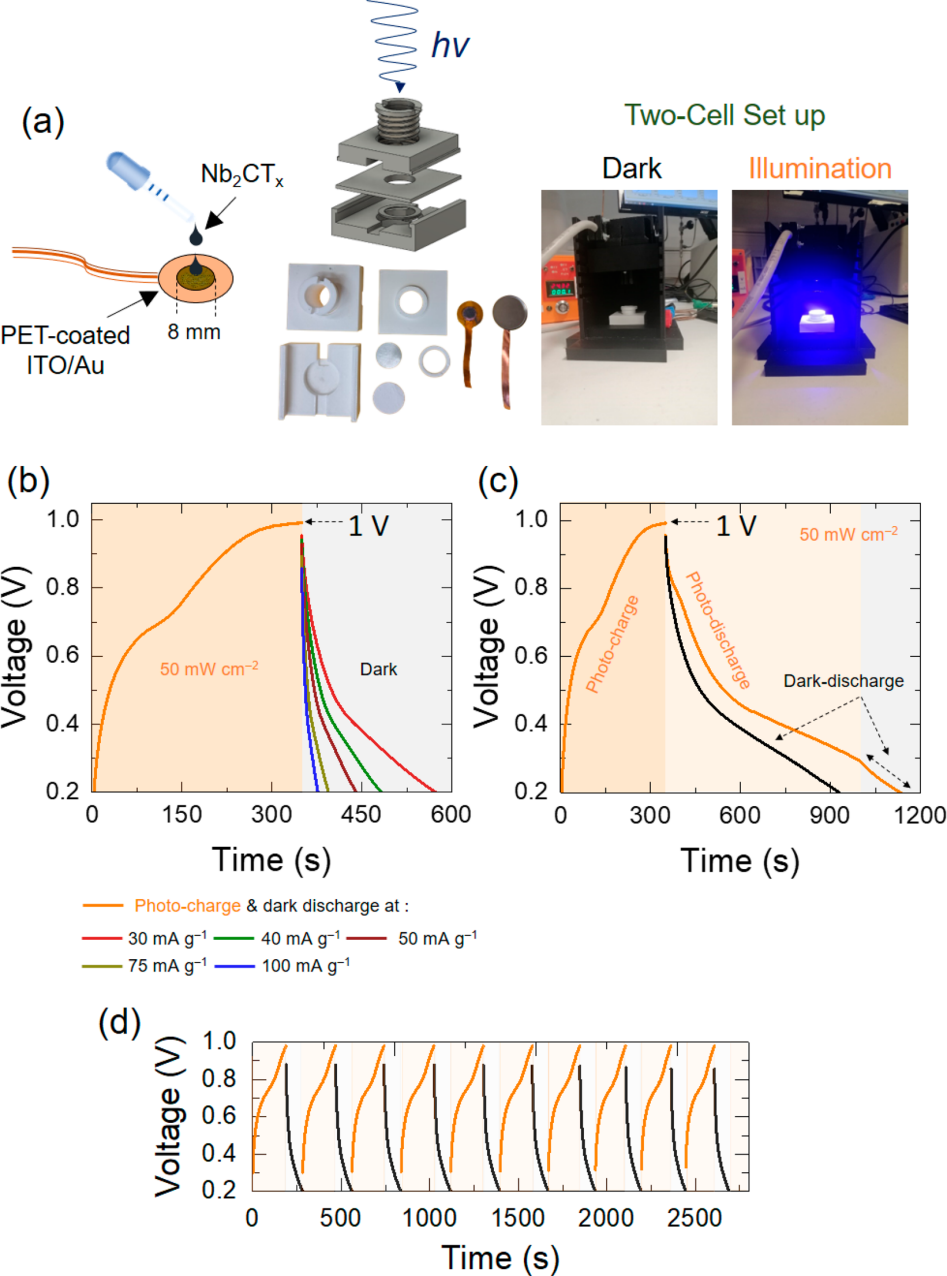
(a) Schematic illustration of the preparation of Nb_2_CT_*x*_-based photocathode and the two-electrode cell setup under dark and illumination conditions. (b) Photocharged (λ = 435 nm, 50 mW cm^–2^, at 0.02 mA cm^–2^) and dark-discharged (at different specific current rates) of the Nb_2_CT_*x*_-based P-ZIC. (c) Photocharged and photodischarged (λ = 435 nm, 50 mW cm^–2^, at 0.02 mA cm^–2^) and dark-discharged (at 0.02 mA cm^–2^) Nb_2_CT_*x*_-based P-ZIC. (d) Cyclic photocharged (λ = 435 nm, 50 mW cm^–2^, at 0.03 mA cm^–2^) and dark-discharged (at 0.06 mA cm^–2^) behavior of the Nb_2_CT_*x*_-based P-ZIC in its initial 10 cycles.

Initially, dark- and photocurrent efficiency of the Nb_2_CT_*x*_-based P-ZIC was analyzed using light-dependent chronoamperometry in dark and illumination conditions. Three different LEDs of λ = 435 nm [intensity of 25 and 50 mW cm^–2^ at 0, 0.2, 1, and open circuit potential (OCP) applied voltages, OCP = 0.93 V], λ = 533 nm (intensity of 50 and 100 mW cm^–2^ at 0 applied voltage), and λ = 630 nm (intensity of 50 and 100 mW cm^–2^ at 0 applied voltage) were employed for this purpose (Figures S1 and S2). During the OCP conditions for photocharging, the movement of electrons through the external circuit is driven by the internal electric field established by the built-in potential difference within the anode and photocathode materials of the hybrid zinc-ion capacitor. This process allows for the maintenance of charge separation and the increase in the degree of OCP across the terminals. It is distinguished that the maximum photocurrent response (Δ*I* = *I*_light_ – *I*_dark_) was for λ = 435 nm. Hence, λ = 435 nm was selected for the next characterization to determine the performance of the P-ZIC.

Next, the P-ZIC was photocharged with a light intensity of 50 mW cm^–2^, a low current density of 0.02 mA cm^–2^, at the voltage range of 0.2–1.0 V, and then discharged by galvanostatic process at different specific current rates (Figure 3b). That specific voltage range was optimized to avoid any dehydration of electrolyte ions by hydrogen evolution reduction and oxygen evolution reactions (Figure S3). It can be seen that the photocharging voltage response of the cell reaches the cutoff voltage of 1.0 V after ∼350 s, which is greater than the most recent reports of photoenhanced capacitors (Table S1). To assess the photocharge, photodischarge, and dark-discharge behavior of the cell in further detail, the P-ZIC was charged under illumination and then discharged at two situations of illumination and dark with the same current densities (0.02 mA cm^–2^) (Figure 3c). The P-ZIC gains an advantage from a constant flow of photons throughout the photocharge and photodischarge states, which facilitate a continuous generation of electron–hole pairs. As a result, the charge transfer is accelerated with faster charging compared with the discharging when light energy continues to generate electron–hole pairs. However, the photovoltaic influence stays passive in the dark mode with no ongoing creation of electron–hole pairs. Hence, the discharge process of the capacitor relies entirely on the stored charge process. Figure S4 also depicts the photocharging of the P-ZIC under a voltage-floating condition with a very low current density of 0.006 mA cm^–2^ where it attains ∼0.96 V after ∼1000 s. The initial cycling stability of the P-ZIC was also examined by applying 10 repeated photochargings (50 mW cm^–2^, 0.03 mA cm^–2^) and galvanostatic dischargings (0.06 mA cm^–2^) (Figures 3d). A steady-state photocharge and galvanostatic discharge activity was detected during the cycling.

Cyclic voltammetry (CV) and galvanostatic charge–discharge (CD) characterizations were performed on the P-ZIC under dark and illumination (25 and 50 mW cm^–2^) conditions at different scan rates to estimate the energy storage performance. The CVs showed pseudocapacitive features with intercalation and partial oxidation–reduction (redox) (Figure 4a–d and Figure S5a,b). This energy storage mechanism is similar to a battery-type intercalation process^32^ with a difference in fast reaction kinetics specific to a supercapacitor electrode. The redox peaks can refer to the reversible redox reaction that is accomplished at the Zn anode during charge–discharge cycles with the statement that Zn^2+^ ions are removed from the anode and transferred to the electrolyte while releasing 2e^–^ throughout charging. Concurrently, there will not be any typical redox reaction in the cathode except SO_4_^–2^ adsorption and electrostatic storage.

**Figure 4:**
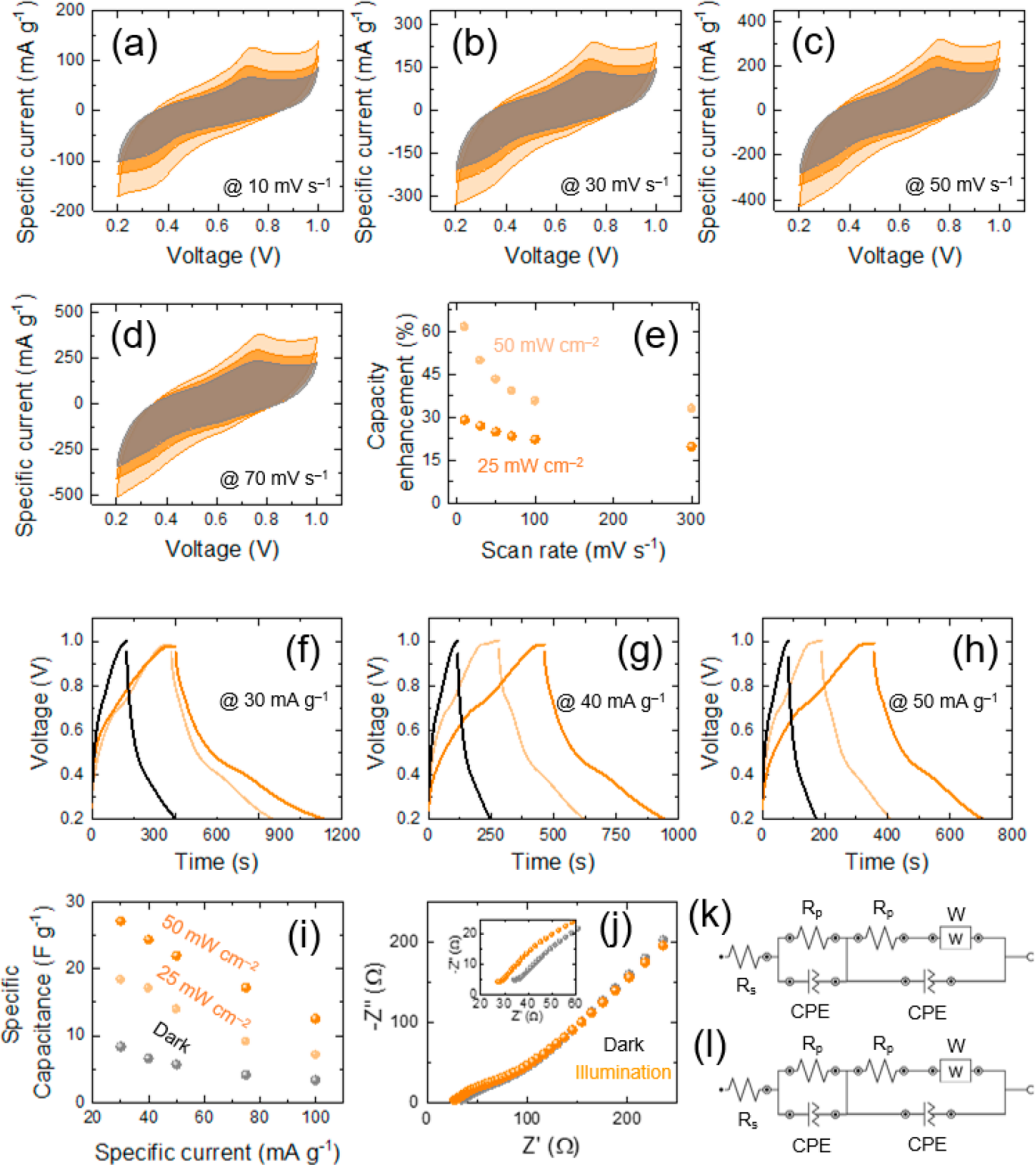
(a–d) Comparative CV analyses of the Nb_2_CT_*x*_-based P-ZIC at different specified scan rates under dark, 25 mW cm^–2^ (dark orange), and 50 mW cm^–2^ (light orange) (λ = 435 nm) illumination conditions. (e) Diagram of the capacity enhancement under illumination (λ = 435 nm) with 25 and 50 mW cm^–2^ intensities versus different scan rates. (f–h) Comparative CD analyses of the Nb_2_CT_*x*_-based P-ZIC at different identified specific currents under dark, 25 mW cm^–2^ (light orange), and 50 mW cm^–2^ (dark orange) illumination (λ= 435 nm) conditions. (i) Comparative specific capacitance at different specific currents under dark and illumination conditions with specified light intensities on the figure. (j–l) Nyquist plots and equivalent circuit diagrams of the Nb_2_CT_*x*_-based P-ZIC in dark and illumination (λ = 435 nm, 50 mW cm^–2^) conditions.

A reverse redox reaction occurs during discharge with the movement of Zn^2+^ back onto the anode and release of the stored SO_4_^–2^ from the cathode into the electrolyte to complete the cycle. These redox reactions in the anode, along with the cathode’s electrostatic behavior and photogenerated electrons and holes, contribute to the energy storage capabilities of P-ZIC. It can be seen from CV results that light in both applied intensities effectively enhance the storage efficiency of the P-ZIC. The highest capacity enhancement calculated according to equation  (*C*_light_ and *C*_dark_, specific capacitances in illumination and dark, respectively) reaches above 60% with 50 mW cm^–2^ light intensity at the rate of 10 mV s^–1^ (Figure 4e). This capacity enhancement under illumination can be ascribed to the significant separation of photogenerated electrons and holes. This separation notably enhances the conductivity of the electrode materials, which leads to a substantial increase in the charge transport density and storage during the electrolytic process. Figure 4e also indicates the impact of light intensity, thereby revealing that the capacity enhancement diminishes as the light intensity decreases to a lower level. The figure also highlights that capacity enhancement is reduced at the higher scan rates most probably due to insufficient time for the generation of larger electron and holes, as well as limited electrolyte ion diffusion. The CD characteristic behavior at different specific currents (Figure 4f–h and Figure S5c–f) is also in line with the CV results where it can be viewed that light enhances the charge and discharge times because of the generation of photoelectrons. Specific capacitance enhancement at different specific currents (Figure 4i) shows that the specific capacitance of P-ZIC overtakes ∼27 F g^–1^ with 50 mW cm^–2^ light intensity at 30 mA g^–1^ specific current, which is almost 3 times larger than under dark condition and 1.5 times greater than under illumination status with 25 mW cm^–2^ intensity. To explore the characteristics of charge transport and ion diffusion, the Nyquist impedance spectra of the P-ZIC in dark and illumination (50 mW cm^–2^) circumstances were first examined (Figure 4j) and fitted (Figure S6), and then their Randles circuits were simulated with very low errors of χ^2^ = 0.01 (dark) and χ^2^ = 0.02 (illumination) (Figure 4k,l). It is seen (Figure S6) that the P-ZIC has a lower resistance (*R* = ∼27 Ω) under illumination than with the dark position (*R* = ∼29.6 Ω). This is evident from the shift of the overall resistance toward lower values during illumination because of larger photogenerated charge carriers. As stated by the fitted results, each circuit possesses an electrolyte solution resistance (*R*_s_) in series with a parallel blend of the charge transfer resistance (*R*_p_) and constant phase element (CPE). The second part of each Randles circuit has an extra impedance (*W*) representing a Faradaic reaction in addition to the previous elements. The impedance defines the ion diffusion, transport, and kinetic processes within the electrolyte.

The energy density, power density, and operation mode performance summary of the Nb_2_CT_*x*_-based P-ZIC were calculated in dark and illumination conditions at five different specific currents (Figure 5a–c). It is observed that the energy density values are enhanced under illumination because of larger photogenerated charges and specific capacitance. However, the power density under illumination will remain at the same value in dark conditions. This effect corresponds to the discharge time value with light intensity. Once the light intensity increases, the discharge time value also will be enhanced with the same proportion because of the already vast storage charges. Hence, the power density [*P* = (*E* × 3600)/*t*, *E* = energy density, and *t* = discharge time) will remain the same. The long-term capacity retention and Coulombic efficiency of Nb_2_CT_*x*_-based P-ZIC were determined over 3000 cycles at a specific current of 100 mA g^–1^ in the dark status (Figure 5d). The graph shows that the sample provides an almost stable Coulombic efficiency. However, the capacitance retention reaches ∼85% after 3000 cycles. Morphology and EDS characteristics of the Nb_2_CT_*x*_-based photocathode after stability were evaluated to find a reason for the ∼15% reduction (Figure S7a). The EDS results demonstrate a trace of sulfur and zinc relevant to the residual electrolyte and a large peak of fluorine due to the Nafion. Those residual zinc and sulfur elements probably diminish the stability of the photocathode. A set of CV characterizations at a scan rate of 10 mV s^–1^ and CD at a specific current of 75 mA g^–1^ (Figure S7b,c) were conducted on the P-ZIC in dark and illumination (50 mW cm^–2^) conditions to examine photoenhancement efficiency of the device after stability test. The findings indicate that even though the photoenhancement efficiency of the P-ZIC diminishes after stability, it continues to play an efficient role in responding to light stimuli throughout the charge and discharge processes.

**Figure 5:**
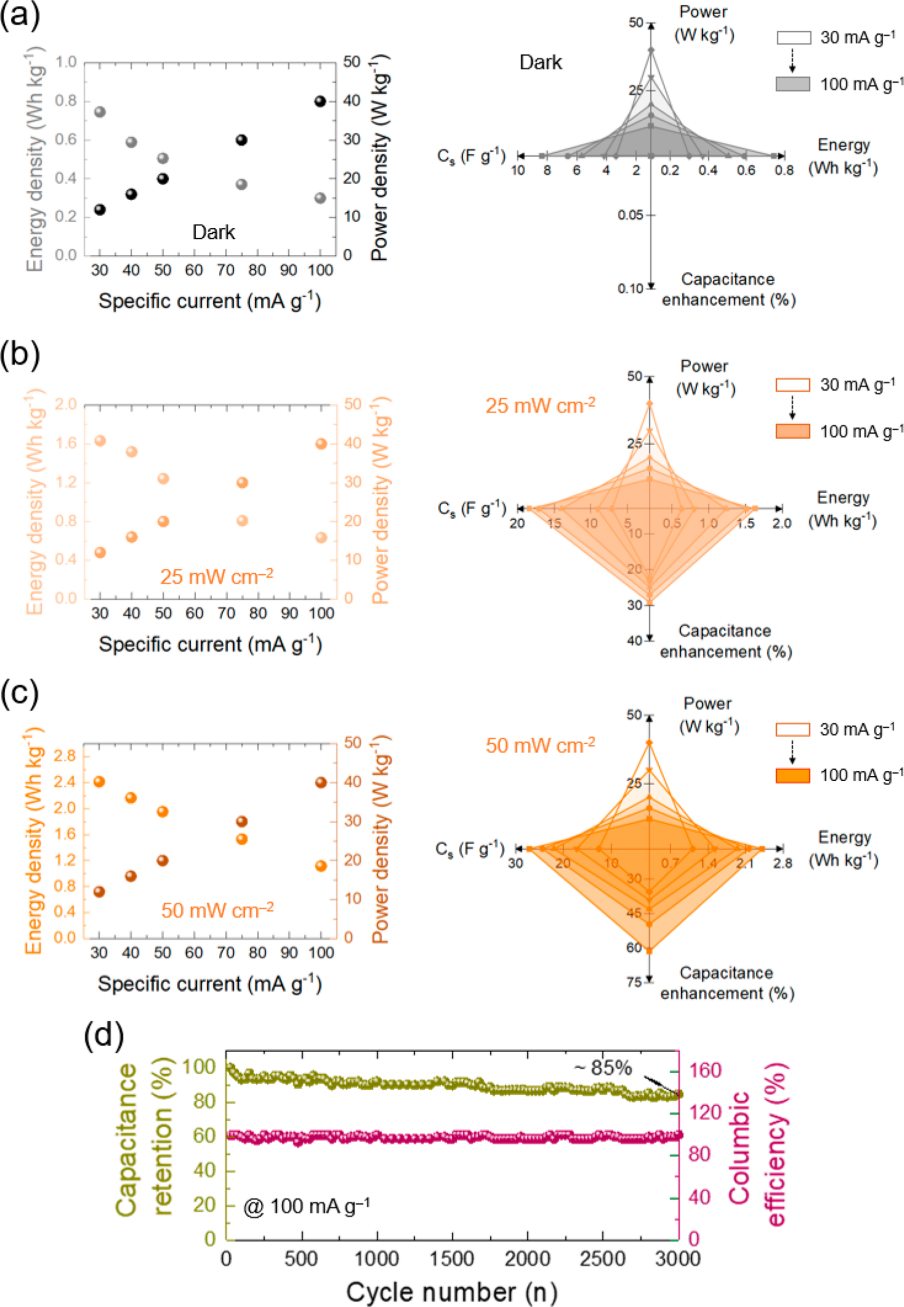
Calculated energy and power densities, along with performance summary of operation modes at different specific currents under (a) dark, (b) illumination (λ = 435 nm) at 25 mW cm^–2^, and (c) illumination (λ = 435 nm) at 50 mW cm^–2^ conditions on Nb_2_CT_*x*_-based P-ZIC. (d) Long-term cyclic stability of the Nb_2_CT_*x*_-based P-ZIC after 3000 cycles.

In summary, this research presents a groundbreaking exploration into the development of dual-functional photoactive cathodes by introducing a novel approach to leverage MXene-based materials for this purpose. The study highlights the effectiveness of a Nb_2_CT_*x*_-based photocathode in P-ZIC, which enables direct charging through light absorption and eliminates the need for external photovoltaic devices. The evaluation involved comparing the efficiency of the Nb_2_CTx-based P-ZIC to two distinct low-light intensities of 25 and 50 mW cm^–2^ with λ = 435 nm. The results exhibit a capacitance enhancement of over 60% under an intensity of 50 mW cm^–2^. Moreover, this achievement presents an impressive output voltage of 1.0 V at the same light intensity. Further analysis of the fabricated P-ZIC also revealed a very good capacitance retention rate of ∼85% after undergoing 3000 charge–discharge cycles. This study anticipates the potential of MXene nanomaterials to not only provide a powerful single-architecture platform for new compact off-grid energy storage devices but to also be a perspective to expand additional new MXene-based nanostructures by their suitable surface chemistry modification for the next-generation flexible photoenhanced energy storage devices. This anticipation is because of the mass production advantage of MXenes and their remarkable intrinsic properties. The characteristics have the potential to enable the manufacture of a simple and efficient device architecture compared with existing alternatives with integrated energy harvesting and energy storage segments.

## Supporting Information Available

Additional experimental details, sample preparation, and characterization methods; cyclic current response for all applied lights with different intensities; CV analysis of Nb_2_CT_*x*_-based P-ZIC at the voltage range of −0.3 to 1.2 V and its optimized range; voltage floating analysis under continuous illumination; comparative CV and CD curves at different scan rates and specific currents under dark and illumination, as well as comparative charge and discharge time at different specific currents under dark and illumination; the fitted electrochemical impedance spectroscopy (EIS) of the P-ZIC in dark and illumination conditions and the different fitted parameters of Randles circuits; SEM, EDS, CV, and CD analyses of Nb_2_CT_*x*_-based photocathode of the P-ZIC after 3000 life cyclings; and comparative characteristics of some cutting-edge photoenhanced supercapacitors and hybrid capacitors (PDF)
