# Supplementary material for: Decoding Niobium Carbide MXene Dual-Functional Photoactive Cathode in Photoenhanced Hybrid Zinc-Ion Capacitor
Source: ACS Mater Lett. 2024 Mar 8;6(4):1338–46. doi: 10.1021/acsmaterialslett.3c01661 (PMC10988777; doi:10.1021/acsmaterialslett.3c01661)
Supplement: Supplementary file 1 — tz3c01661_si_001.pdf [file tz3c01661_si_001.pdf]

Supporting Information

Decoding Niobium Carbide MXene Dual-Functional Photoactive Cathode in Photo-Enhanced Hybrid Zinc-Ion Capacitor

Jalal Azadmanjiri*, Jakub Regner, Jiri Sturala, Zdeněk Sofer*

Department of Inorganic Chemistry, University of Chemistry and Technology Prague, Technická 5, 166 28 Prague 6, Czech Republic

*Corresponding Authors: jalal_azad2000@yahoo.com, jalal.azadmanjiri@vscht.cz (Jalal Azadmanjiri); zdenek.sofer@vscht.cz (Zdeněk Sofer)

Experimental

Sample and photo-E ZIC cell preparation

Synthesis of Nb_2CT_x MXene: 40 g of as-received Nb_2AlC MAX material (Jinzhou Haixin Metal Materials, China) was added portion-wise to 600 mL ice-cold hydrofluoric acid (HF) solution (50% vol, Sigma-Aldrich) and continuously stirred under ambient conditions for two days. The mixture was then diluted with distilled water and decanted three times. Following this, a mixture of hydrochloric acid (HCl, 400 mL of 35% vol), lithium fluoride (LiF, 40 g), and 100 mL distilled water was added to the wet product obtained from the previous step and stirred at room temperature for 3 days before it was diluted with 2 L distilled water and decanted three times. After that, a mixture of 400 mL 50% HF and 300 mL 35% HCl was added to the wet product and stirred again for another 3 days, diluted with 2 L distilled water, and decanted three times. Next, the obtained product was mixed with 400 ml distilled water and 200 ml tetramethylammonium hydroxide ($C_4H_{13}NO$, 25% vol), and stirred for three days. The reaction mixture was diluted with 2 L distilled water and decanted three times. The final product was diluted with 500 mL distilled water shaken for 2 minutes and repeatedly centrifuged approximately four times until a neutral pH reaction. In the end, the product was freeze-dried and stored in the argon-filled glovebox.

Preparation of the Nb_2CT_x-based photocathode: A flexible and transparent polyethylene terephthalate-coated indium tin oxide (80 nm) and gold (20 nm) (PET-coated ITO/Au) substrate, with a circular shape and a diameter of 15 mm, was chosen as a substrate to drop-cast the synthesized Nb_2CT_x-based slurry. 20 nm Au coating was to enhance the conductivity of the transparent PET-coated ITO substrate and make the ITO more stable during the next steps of characterization. Prior to the drop-cast of the Nb_2CT_x MXene sample on PET-coated ITO/Au, 40 mg (80 wt%) of the synthesized Nb_2CT_x MXene was mixed with 10 wt% carbon black (Super P,

99%) and 10 wt% polyvinylidene fluoride [PVDF, Alfa Aesar, Thermo Fisher Scientific] in 2 mL N-Methyl-2-pyrrolidone (NMP, 99%, Acros Organics, Thermo Fisher Scientific) solvent. Then, the mixture was sonicated for at least 30 minutes and kept under stirring (250 rpm) overnight to obtain a homogeneous slurry. Afterward, 10 μL of the slurry was drop-casted on the PET-coated ITO/Au substrate and dried in a vacuum oven at 35 °C for 24 hours.

Design and fabrication of the photo-E ZIC cell: The as-prepared photocathode, pre-cut circle-shaped Zn foil anode with a thickness of 1 μm thickness and 15 mm diameter, a circle-shaped Whatman glass microfiber filter paper separator with 18 mm diameter, and a 2016-type coin cell positive case with ~ 50 μl of 2 M $ZnSO_4$ aqueous electrolyte were all assembled inside a printed holder with polylactic filament PLA (Prusa polymers). An optical window with an 8 mm diameter was applied to the printed holder for illumination. To perfectly seal the cell, a square-shaped sheet (1 mm thickness) with a hole in its center (8 mm diameter) was also printed and slid onto the assembled capacitor cell to secure it completely.

Characterization methods

Materials characterization: The crystal structure and phase exploration of the synthesized Nb_2CT_x MXene was identified by X-ray diffraction (XRD, Bruker D8 Discoverer, Germany) with Cu Kα radiation (λ = 0.15418 nm, U = 40 kV, I = 40 mA) and 2θ degree (2θ = 5–90°) with a step size of 0.02°. Raman spectroscopy characterization was also conducted on the synthesized Nb_2CT_x MXene using a Raman microscope (Renishaw, England) in backscattering geometry with a charge-coupled device detector. To do so, the synthesized Nb_2CT_x MXene was deposited on a metal plate as a powder and measured directly after the deposition. A diode-pumped solid-state green laser (532 nm) with a low incident power of 0.5 mW, exposure time of 30 s, and 50×

objective lens was applied to the sample during characterization. The reference point for calibration of the Raman instrument was a silicon peak position at 520 cm^{-1}. The morphology and elemental mapping of the synthesized Nb_2CT_x MXene and its slurry was performed by scanning electron microscopy coupled with energy-dispersive X-ray spectroscopy (SEM/EDS, Tescan Lyra 3).

Electrochemical characterization: In order to get spatially-resolved insights into the conductivity and catalytic activity of the Nb_2CT_x-based slurry at the microscale, scanning electrochemical microscopy (SECM, Sensolytics, Germany) was adopted as a robust analytical approach. Assessment of the surface and conductivity characteristics of the slurry was made by feedback mode of SECM. An ultramicroelectrode (UME) systematically approached the sample surface at each scan point of the 4D array scan from a solution upper the targeted area. It monitored variations in Faraday current that can be influenced by factors such as surface reactivity, roughness, the distance between the electrode and surface, UME size, and the geometry of the diffusion region. A platinum UME with a 5 μm diameter was employed as a working electrode to scan the materials' surface, focusing on a specific 150 μm^2 area with 2.5 μm increments in both of x and y directions. A platinum wire also served as the counter electrode. To prepare the sample for SECM analysis, approximately 10 μL of the Nb_2CT_x-based slurry was dropped inside a generated cavity with a dimension size of 100 μm. The cavity was prepared by photolithography and wet etching on a pre-cleaned silicon wafer and dried in a vacuum oven before testing. The SECM analysis was conducted within a potassium ferrocyanide redox system solution ($K_4[Fe(CN)_6]$), containing 10 mmol L^{-1} of ferrocyanide ($[Fe(CN)_6]^{4-}$) as a redox mediator and 0.1 mol L^{-1} of potassium chloride (KCl) as a supporting electrolyte. The potential of UME was set at E=0.5 V versus the Ag/AgCl

reference electrode to drive the redox reaction with the conversion of $Fe(CN)_6^{4-}$ to $Fe(CN)_6^{3-}$ and the transfer of electrons ($Fe(CN)_6^{4-} \rightarrow Fe(CN)_6^{3-} + e^-$) until it gains a steady state.

Electrochemical analysis of the photo-E ZIC was conducted using an Autolab PGSTAT 204 (NOVA, Utrecht, Netherlands) under dark and illuminated conditions. The electrochemical efficiency was rigorously evaluated through a range of techniques including cyclic voltammetry (CV), galvanostatic charge-discharge (CD), electrochemical impedance spectroscopy (EIS), and chronoamperometry. CV and CD measurements were tested at varying scan rates of 10, 30, 50, 70, 100, and 300 mV s^{-1}, and specific currents of 30, 40, 50, 75, and 100 mA g^{-1}. These investigations were carried out within the optimized voltage window of 0.2–1.0 V. EIS assessments were carried out in dark and illumination conditions with an open circuit potential at a voltage amplitude of 5 mV in the frequency range of 10 mHz to 100 kHz. Chronoamperometry measurements were conducted at 0, 0.2, and 1 applied voltage as well as at open circuit potential (OCP) using different light emitting diode (LED) sources of 435 nm (violet), 533 nm (green), and 630 nm (red) wavelengths under 25, 50 and 100 mW cm^{-2} illuminations. These experiments aimed to assess the photocurrent responsivity of the samples under varying light sources. In addition, a three-electrode system was also established to gauge the capacitance and photocurrent responsivity of the sample with the same potentiostat instrument. In this setup, 10 µl of Nb_2CT_x-based slurry was deposited on PET-coated ITO/Au substrate, serving as a working electrode, while platinum (Pt) wire and $Hg(l)/Hg_2Cl_2(s)/KCl(sat)$ were employed as counter and reference electrodes, respectively. CV scans within three-electrode analysis spanned a voltage window from 0.0–0.5 V with scan rates of 5–100 mV s^{-1}. To explore the photocurrent responsivity of the Nb_2CT_x-based sample at zero applied voltage, chronoamperometry characterization was carried out under illumination using a 435 nm wavelength LED source. Furthermore, the unique responses of the

Nb_2CT_x-based sample to illumination were investigated using linear sweep voltammetry (LSV) at a scan rate of 5 mV s^{-1} under dark and illumination conditions with light intensity of 100 mW cm^{-2}. The maximum temperature generated in the hybrid zinc-ion capacitor device during illumination could belong to the applied high-energy LED with a wavelength of 435 nm and the maximum proposed intensity of 100 mW cm^{-2}. The temperature can be estimated using the Stefan-Boltzmann law and the relationship between intensity and temperature in the context of radiative heat transfer.[1] The Stefan-Boltzmann law is expressed as: $P = \sigma . A . \varepsilon . T^4$, where; P is the power emitted per unit area (in watts per square meter), σ is the Stefan-Boltzmann constant (5.67×10^{-8} W m^{-2} K^{-4}), A is the surface area, ε is the emissivity, and T is the absolute temperature. Given that intensity (I) is the power per unit area and is related to power (P) by the formula $I = P/A$, the Stefan-Boltzmann Law can be rewritten in terms of intensity: $I = \sigma . \varepsilon . T^4$.

Defined the values I = 100 mW cm^{-2}, $\sigma = 5.67\times10^{-8}$ W m^{-2} K^{-4}, and emissivity, the accurate temperature generated in the hybrid zinc-ion capacitor can be obtained. According to the fact that the PET degradation temperature is in the range of 270-370 °C,[2] and the stability of Nb_2CT_x MXene is ~ 800 °C,[3] as well as low visual observed of generated temperature during the analysis, the hybrid zinc-ion capacitor device could be stable during the illumination with wavelengths of 435, 533 and 630 nm, and intensity of 25, 50 and 100 mW cm^{-2}.

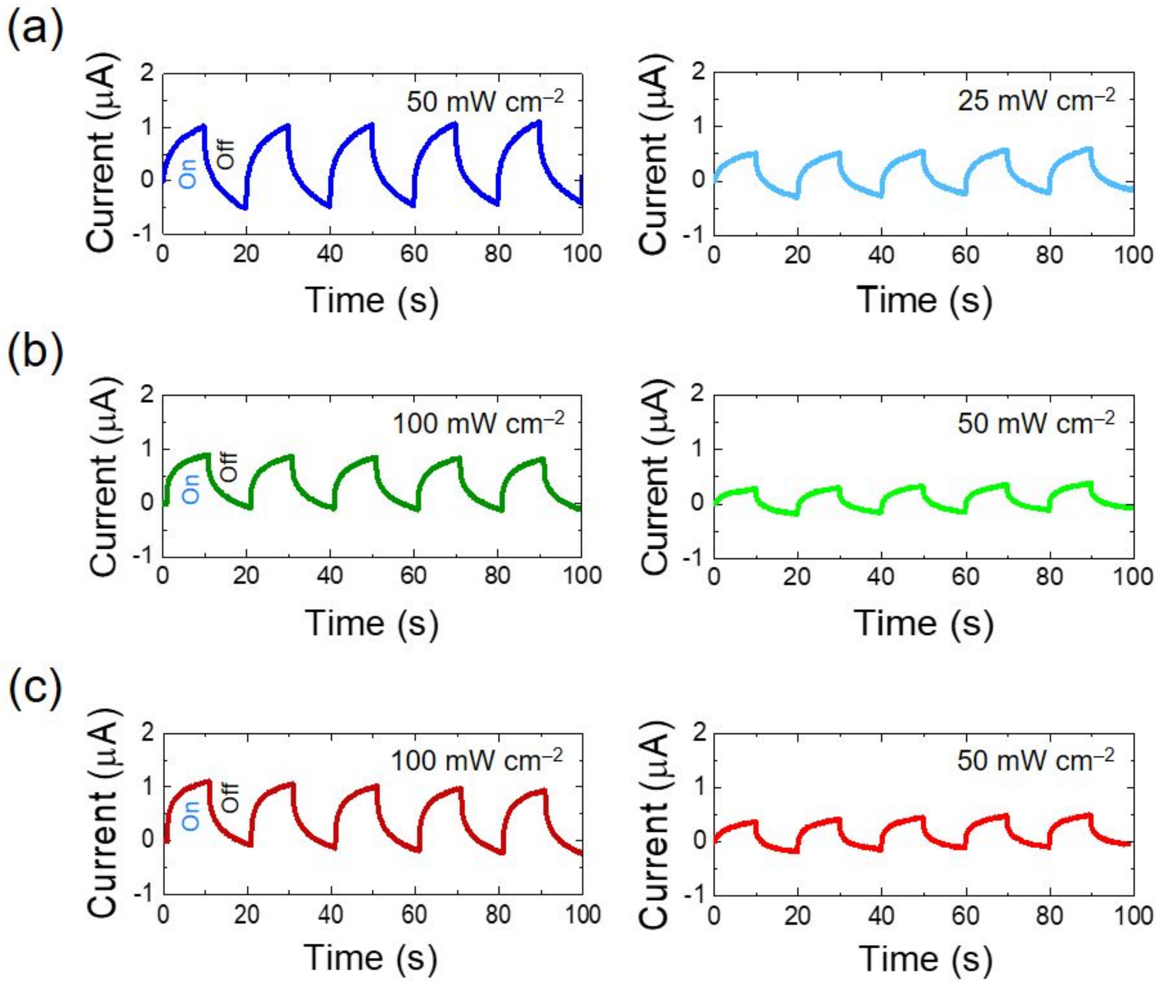

Figure S1. Cyclic current response ($\Delta I = I_{light\ (on)} - I_{dark\ (off)}$) of the Nb_2CT_x-based photo-E ZIC using different LED lights of (a) λ= 435 nm (25 and 50 mW cm^{-2}), (b) λ= 533 nm (50 and 100 mW cm^{-2}), and (c) λ= 630 nm (50 and 100 mW cm^{-2}), at zero applied voltage. (Note: $I_{dark\ (off)}$ and $I_{light\ (on)}$ stand for the currents in dark and illuminated conditions).

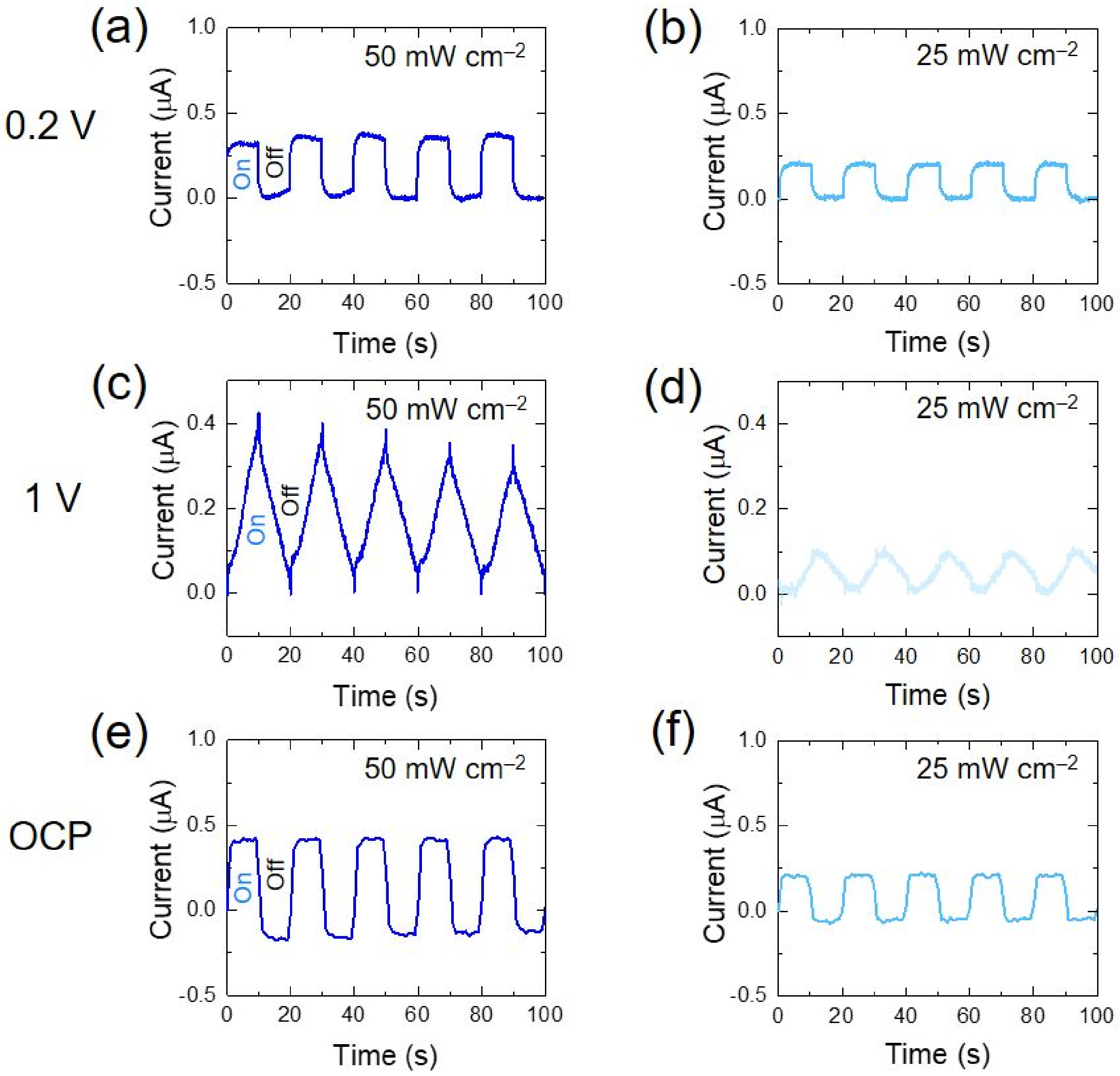

Figure S2. Cyclic illumination (λ= 435 nm, intensities of 25 and 50 mW cm^{-2}) and dark current response behaviors of the Nb_2CT_x-based photo-E ZIC at (a and b) 0.2 V, (c and d) 1 V, and (e and f) OCP (0.93 V). Note: the current response in the y-axis is equal to $\Delta I = I_{light\,(on)} - I_{dark\,(off)}$.

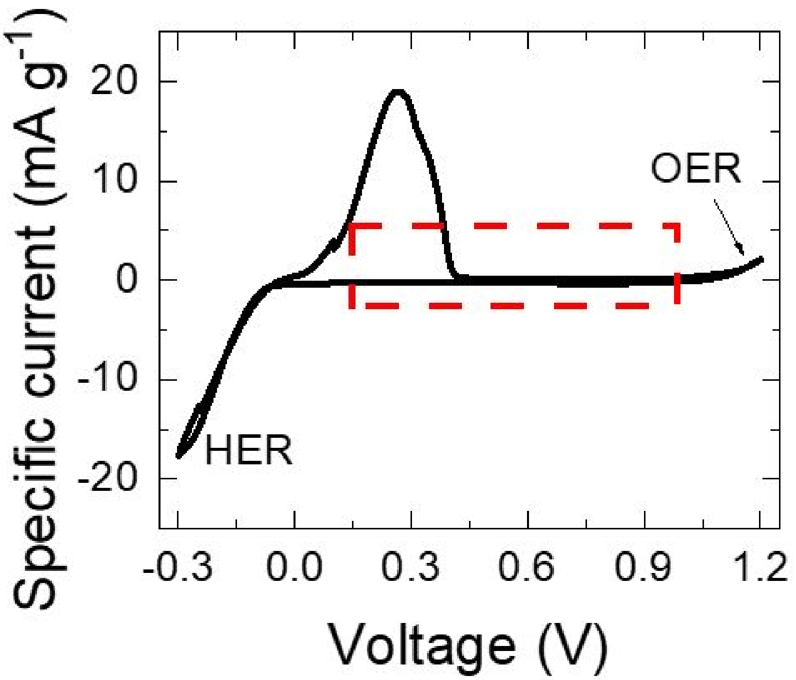

Figure S3. CV analysis of Nb_2CT_x-based photo-E ZIC at the voltage range of -0.3 to 1.2 V (black) at a scan rate of 10 mV s^{-1}, and optimized CV range (red area).

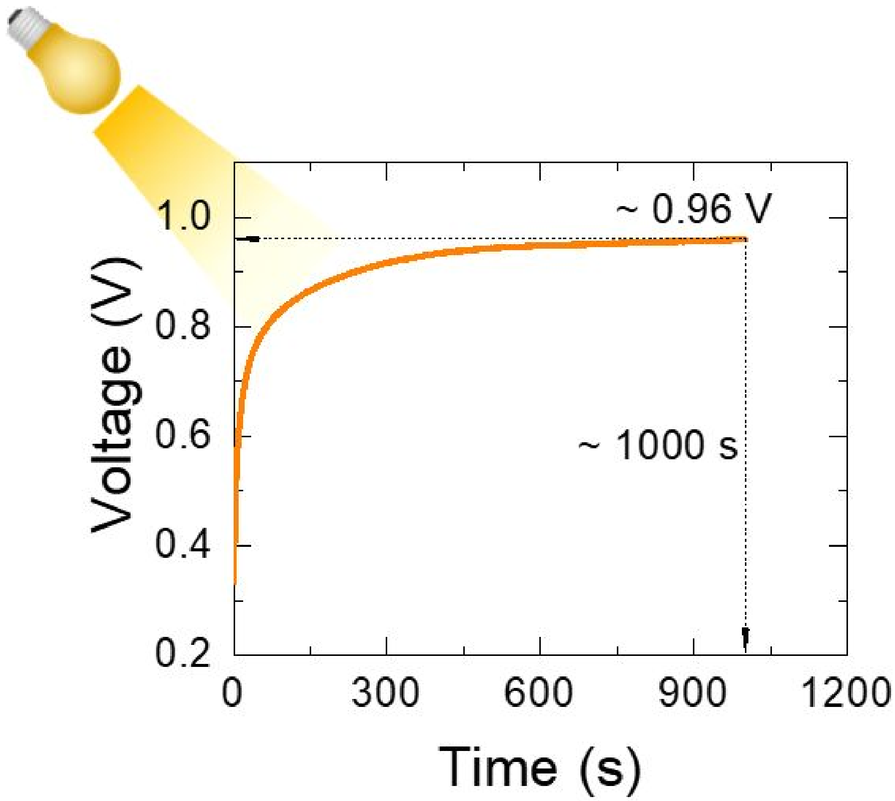

Figure S4. Voltage floating analysis under continuous illumination (λ = 435 nm, 50 mW cm^{-2}) at a very low specific current of 0.006 mA cm^{-2} on photo-E ZIC.

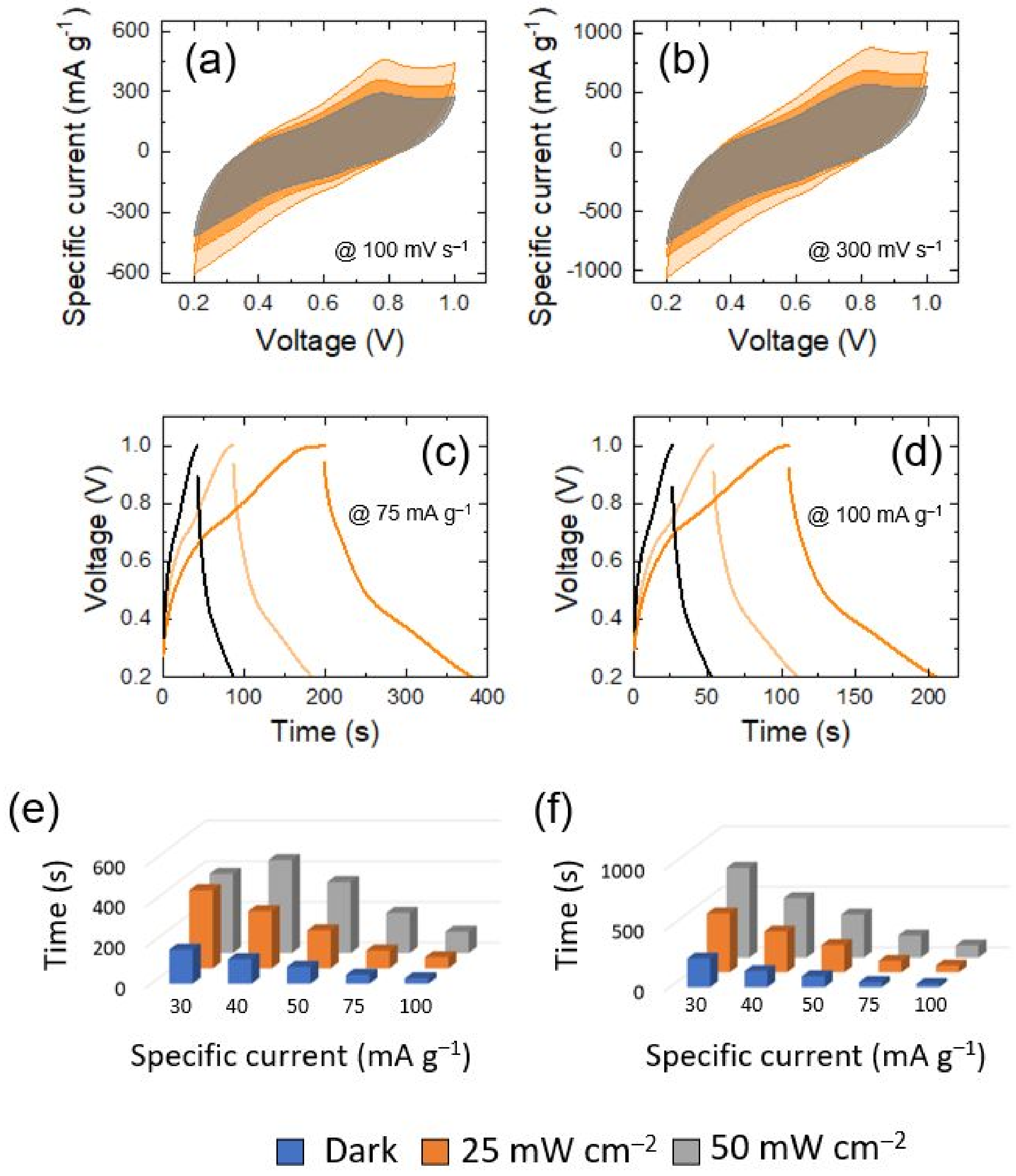

Figure S5. (a and b) comparative CV curves at scan rates of 100 and 300 mV s^{-1} under dark, 25 mW cm^{-2} (dark orange), and 50 mW cm^{-2} (light orange) illumination conditions. (c and d) comparative CD curves at specific currents of 75 and 100 mA g^{-1} under dark, 25 mW cm^{-2} (light orange), and 50 mW cm^{-2} (dark orange) illumination conditions. Comparative (e) charge time and (f) discharge time at different specific currents under dark and illumination with 25 mW cm^{-2} and 50 mW cm^{-2}. All CVs and CDs were done with λ = 435 nm for Nb_2CT_x-based photo-E ZIC.

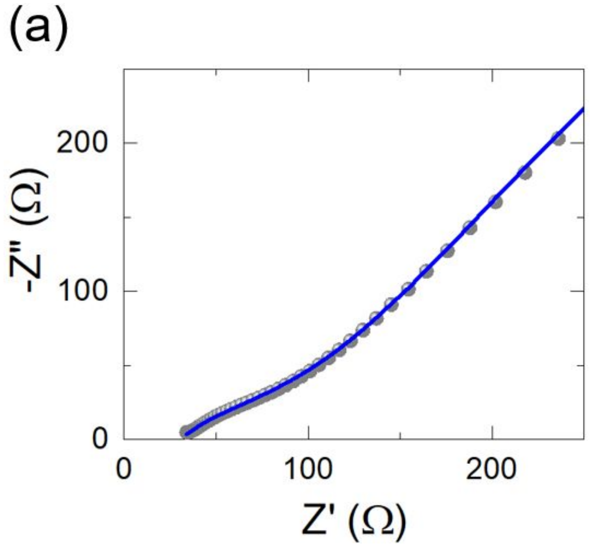

Dark		Illumination	
R_s	29.6 Ω	R_s	27.0 Ω
R_p	166 Ω	R_p	48.8 Ω
CPE.Y0	Y0 = 369 μMho×S^N N = 0.461	CPE.Y0	Y0 = 79.1 μMho×S^N N = 0.646
R	40.1 Ω	R	10.8 Ω
W.Y0	Y0 = 463 μMho×$S^{0.5}$	W.Y0	Y0 = 461 μMho×$S^{0.5}$
CPE.Y0	Y0 = 53.9 μMho×S^N N = 1.04	CPE.Y0	Y0 = 97.2 μMho×S^N N = 0.717
χ^2	0.01	χ^2	0.02

Figure S6. The fitted EIS of the photo-E ZIC in (a) dark and (b) illumination conditions. The table also indicates different fitted parameters

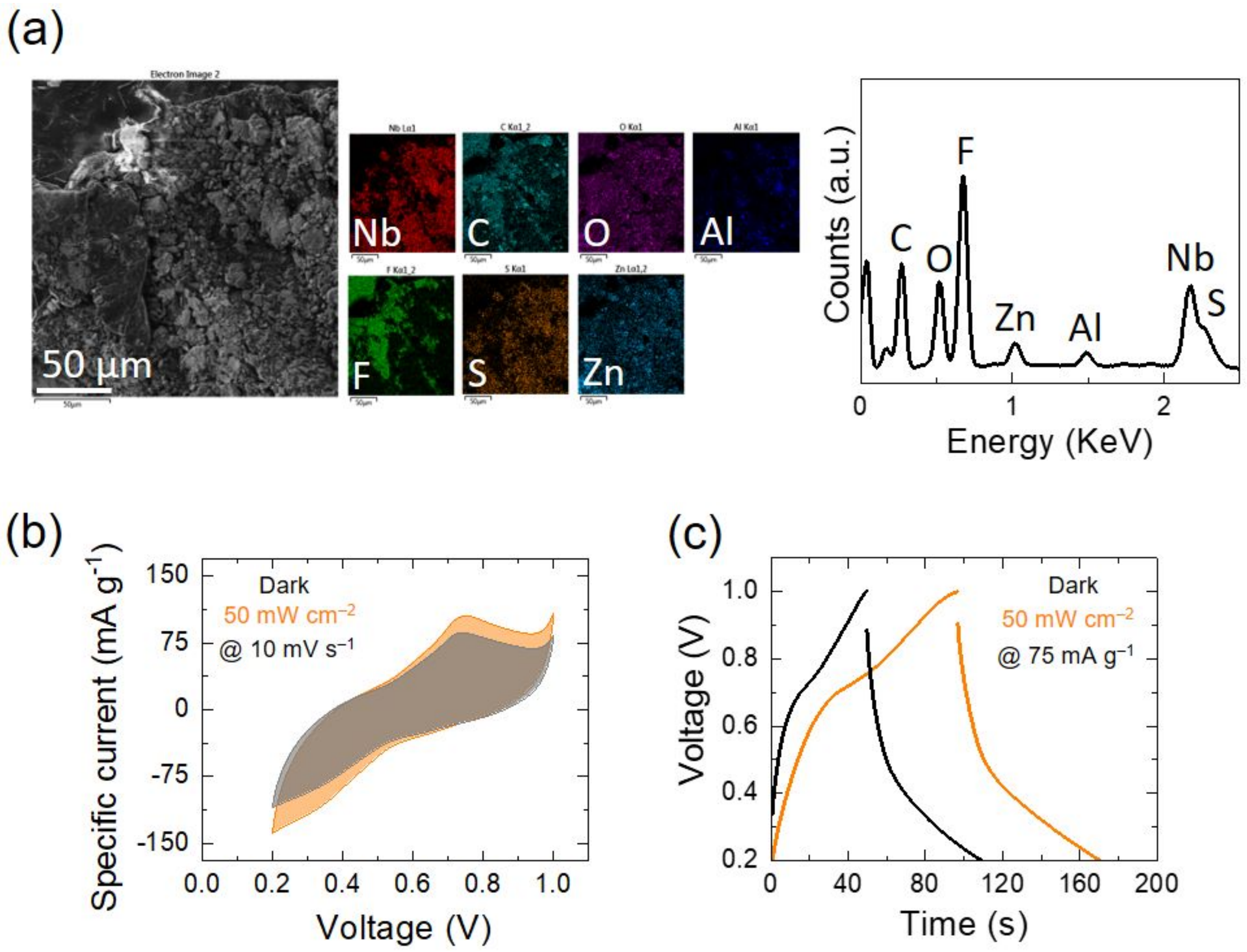

Figure S7. (a) SEM and EDS analyses of Nb_2CT_x-based photocathode, (b) CVs at 10 mV s^{-1} under dark and 50 mW cm^{-2} illumination, and (c) CDs at 75 mA g^{-1} under dark and 50 mW cm^{-2} illumination, for photo-E ZIC after 3,000 life cycling stability.

Photo-rechargeable energy storage system		Electrolyte	Illumination	Specific Capacitance	Energy- and Power- density	Voltage response	Photo enhancement efficiency	Ref.
	Carbon dots modified $Ti_3C_2T_x$-based fibrous supercapacitor	1M H_2SO_4	400 nm ≤ λ ≤ 800 nm, intensity ~ 150 mW cm^{-2}	630 F g^{-1} @ 10 A cm^{-3}	18.75 mWh cm^{-3} and 8382 mW cm^{-3}	~ 400 mV	~ 35.9%	[4]
	Nickel-cobalt-deposited tungsten-doped TiO_2 nanotube supercapacitor	3 M KOH	Visible, intensity ~ 100 mW cm^{-2}	~ 75.2 mF cm^{-2} @ 0.04 mA cm^{-2}	33.93 × 0^{-3} Wh cm^{-2} and 7.54 × 10^{-3} W cm^{-2} @ 0.04 mA cm^{-2}	~ 450 mV	~ 46%	[5]

Table 1. Comparative characteristics of some cutting-edge photo-enhanced supercapacitors and hybrid capacitors with single architecture and the results of this research work.

	$BiVO_4$-V_2O_5@titania nanotubes supercapacitor	3M KCl	Visible, intensity ~ 100 mW cm^{-2}	~ 288 mF cm^{-2} @ 0.12 mA cm^{-2}	0.04 Wh cm^{-2} and 24 × 10^{-5} W cm^{-2} @ 0.23 mA cm^{-2}	~ 150 mV	-	6
	Cu foam-supported CuO_x@$NiCuO_x$	2M KOH	Perfect Light PLS-SXE300, intensity 1.76 W	2.64 F cm^{-2}	~ 8 Wh m^{-2} and ~ 80 W m^{-2}	~ 600 mV	~ 44%	7
	Sulfur-, tungsten-doped TiO_2 nanotube supercapacitor	0.5 M H_2SO_4	λ = 533 nm, intensity ~ 100 mW cm^{-2}	~ 31 mF cm^{-2} @ 0.23 mA cm^{-2}	6.21 Wh cm^{-2} And 399 W cm^{-2} @ 0.23 mA cm^{-2}	~ 400 mV	-	8
	Zinc-ion capacitor using Ag@V_2O_5-activated carbon	3 M $Zn(CF_3SO_3)_2$	λ = 455 nm, intensity ~ 12 mW cm^{-2}	~ 138 F g^{-1}	~ 53.13 Wh kg^{-1} and ~ 36.74 W kg^{-1}	~ 500 mV	~ 63%	9
	ITO/PET (Supercapacitor)	1 M $LiPF_6$ salt in ethylene	Intensity 100 mW cm^{-2}	127 mAh g^{-1} (after 1000 cycles) 1A g^{-1}	~ 40 Wh kg^{-1} and 2000 W kg^{-1}	~ 3 V	~ 8.41%	10

		carbonate and diethyl carbonate (1:1; *v/v*)						
	Zinc-ion capacitor using 2D g-C_3N_4	2M $ZnSO_4$	λ = 420 nm, intensity ~ 50 mW cm^{-2}	~ 11377 mF g^{-1} @ 5 mA g^{-1}	~ 668 mWh kg^{-1} and ~ 1625 mW kg^{-1} @ 5 mA g^{-1}	~ 850 mV	~ 82%	11
	Mg-ion capacitor using VO_2	1 M $Mg(NO_3)_2$	λ = 455 nm, intensity ~ 12 mW cm^{-2}	~ 63.80 F g^{-1} @ 0.65 A g^{-1}	~ 20.5 mAh kg^{-1}	~ 70 mV	~ 56%	12
	Zinc-ion capacitor using $Ti_3C_2T_x$ MXene	2M $ZnSO_4$	λ = 420 nm, intensity ~ 100 mW cm^{-2}	~ 84 F g^{-1} @ 0.2 A g^{-1}	~ 9.4 Wh kg^{-1} and ~ 90 W kg^{-1} @ 0.2 A g^{-1}	~ 960 mV	~ 30%	13
	Zinc-ion capacitor using Te/$Ti_3C_2T_x$ MXene	2M $ZnSO_4$	λ = 420 nm, intensity ~ 100 mW cm^{-2}	~ 73 F g^{-1} @ 0.2 A g^{-1}	~ 8.2 Wh kg^{-1} and ~ 90 W kg^{-1} @ 0.2 A g^{-1}	~ 960 mV	~ 50%	13

	**Zinc-ion capacitor using Nb_2CT_x MXene**	**2M $ZnSO_4$**	**λ = 435 nm, intensity ~ 50 mW cm^{-2}**	**~ 27 F g^{-1} @ 10 mA g^{-1}**	**~ 2.4 Wh kg^{-1} and ~ 40 W kg^{-1} @ 30 mA g^{-1}**	**~ 1000 mV**	**> 60%**	**This work**

References:

(1) Blevin, W.R.; Brown, W.J. A Precise Measurement of the Stefan-Boltzmann Constant. *Metrologia* **1971**, 7, 15−29.
(2) Samperi, F.; Puglisi, C.; Alicata, R.; Montaudo, G. Thermal Degradation of Poly(Ethylene Terephthalate) at the Processing Temperature. *Polymer Degrade. Stability* **2004**, 83, 3−10.
(3) Liu, R.; Li, W. High-Thermal-Stability and High-Thermal-Conductivity $Ti_3C_2T_x$ MXene/Poly(vinyl alcohol) (PVA) Composites. *ACS Omega* **2018**, 3, 2609−2617.
(4) Wang, H.; Cao, J.; Zhou, Y.; Wang, X.; Huang, H.; Liu, Y.; Shao, M.; Kang, Z. Carbon Dots Modified $Ti_3C_2T_x$-Based Fibrous Supercapacitor with Photo-Enhanced Capacitance. *Nano Res.* **2021**, *14*, 3886-3892.
(5) Momeni, M. M.; Navandian, S.; Aydisheh, H. M.; Lee, B.-K. Photo-Assisted Rechargeable Supercapacitors Based on Nickel-Cobalt Deposited Tungsten-Doped Titania Photoelectrodes: A novel self-powered supercapacitor. *J. Power Sources* **2023**, *557*, 232588.
(6) Renani, A. S.; Momeni, M. M.; Aydisheh, H. M.; Lee, B.-K. New Photoelectrodes Based on Bismuth Vanadate-V_2O_5@TiNT for Photo-Rechargeable Supercapacitors. *J. Energy Storage* **2023**, *62*, 106866.
(7) Ren, Y.; Zhu, T.; Liu, Y.; Liu, Q.; Yan, Q. Direct Utilization of Photoinduced Charge Carriers to Promote Electrochemical Energy Storage. *Small* **2021**, *17*, 2008047.
(8) Momeni, M. M.; Aydisheh, H. M.; Lee, B.-K.; Farrokhpour, H.; Najafi, M. Preparation of Photo-Rechargeable Asymmetric Supercapacitors Using S,W-Codoped Titania: Experimental and Theoretical Insights. *J. Alloys Compd.* **2023**, *960*, 170722.
(9) Boruah, B. D.; Wen, B.; Nagane, S.; Zhang, X.; Stranks, S. D.; Boies, A.; De Volder, M. Photo-Rechargeable Zinc-Ion Capacitors using V_2O_5-Activated Carbon Electrodes. *ACS Energy Lett.* **2020**, *5*, 3132-3139.
(10) Li, C.; Cong, S.; Tian, Z.; Song, Y.; Yu, L.; Lu, C.; Shao, Y.; Li, J.; Zou, G.; Rümmeli, M. H.; et al. Flexible Perovskite Solar Cell-Driven Photo-Rechargeable Lithium-Ion Capacitor for Self-Powered Wearable Strain Sensors. *Nano Energy* **2019**, *60*, 247-256.
(11) Boruah, B. D.; Mathieson, A.; Wen, B.; Jo, C.; Deschler, F.; De Volder, M. Photo-Rechargeable Zinc-Ion Capacitor Using 2D Graphitic Carbon Nitride. *Nano Lett.* **2020**, *20*, 5967-5974.
(12) Park, S. K.; Boruah, B. D.; Pujari, A.; Kim, B.-M.; De Volder, M. Photo-Enhanced Magnesium-Ion Capacitors Using Photoactive Electrodes. *Small* **2022**, *18*, 2202785.
(13) Azadmanjiri, J.; Regner, J.; Děkanovský, L.; Wu, B.; Luxa, J.; Sofer, Z. Powering the Future: Unleashing the Potential of MXene-Based Dual-Functional Photoactive Cathodes in Photo-Rechargeable Zinc-Ion Capacitor. *Small* **2023**, 2305972.
